# A Web-Based Transdiagnostic Intervention for Affective and Mood Disorders: Randomized Controlled Trial

**DOI:** 10.2196/mental.8901

**Published:** 2018-05-24

**Authors:** Bogdan Tudor Tulbure, Andrei Rusu, Florin Alin Sava, Nastasia Sălăgean, Todd J Farchione

**Affiliations:** ^1^ Psychology Department West University of Timisoara Timisoara Romania; ^2^ Center for Anxiety and Related Disorders Department of Psychology Boston University Boston, MA United States

**Keywords:** transdiagnostic, anxiety disorders, depressive disorder, cognitive therapy

## Abstract

**Background:**

Research increasingly supports a transdiagnostic conceptualization of emotional disorders (ie applying the same underlying treatment principles across mental disorders, without tailoring the protocol to specific diagnoses), and many international researchers are currently investigating this issue.

**Objective:**

The aim of this study was to evaluate the efficacy and acceptability of a Web-based transdiagnostic program using a sample of Romanian adults diagnosed with anxiety and/or depression.

**Methods:**

Volunteer participants registered for the study and completed a series of online self-report measures. Participants who fulfilled basic inclusion criteria on these measures were contacted for a telephone diagnostic interview using the Structural Clinical Interview for Diagnostic and Statistical Manual of Mental Disorders, 4th Edition Axis I Disorders (SCID-I). Enrolled participants were randomized to either the active treatment group (N=69) or the wait-list control group (N=36) using a 2:1 ratio. The transdiagnostic treatment was based on the Unified Protocol for Transdiagnostic Treatment of Emotional Disorders (UP; Barlow et al, 2011) that addresses common underlying mechanisms of anxiety and depression. Participants randomized to the active treatment condition received 10 weeks of Web-based treatment based on the UP. Throughout treatment, graduate students in clinical psychology provided guidance that consisted of asynchronous written communication on a secure Web platform. After the intervention, participants in both study conditions were invited to complete a set of self-report measures and a postintervention SCID-I interview conducted by a different team of graduate students blinded to participants’ group and diagnostic status. Six months later, participants in the active treatment group were invited to complete an online follow-up assessment.

**Results:**

During the intervention, active treatment participants completed on average 19 homework assignments (SD 12.10), and we collected data from 79.0% (83/105) at postintervention and 51% (35/69) at follow-up for self-report measures. Postintervention SCID-I interviews were collected from 77.1% (81/105) participants. Relative to the wait-list control group, the transdiagnostic intervention yielded overall medium to large effect sizes for the primary outcome measures (within-group Hedges *g*=0.52-1.34 and between-group *g*=0.39-0.86), and also for anxiety sensitivity (*g*=0.80), symptom interference (*g*=0.48), and quality of life (*g*=0.38). Significant within-groups effects *only* were reported for the active treatment group on Panic Disorder Severity Scale-Self Report (PDSS-SR, *g*=0.58-0.65) and Yale-Brown Obsessive Compulsive Scale (Y-BOCS, *g*=0.52-0.58).

**Conclusions:**

Insignificant between-group differences for the Y-BOCS and PDSS-SR could be explained by the small number of participants with the associated primary diagnostic (eg, only 3 participants with obsessive compulsive disorder) by the choice of outcome measure (PDSS-SR was not rated among the evidence-based measures) and by the fact that these disorders may be more difficult to treat. However, the overall results suggest that the transdiagnostic intervention tested in this study represents an effective treatment option that may prove easier to disseminate through the use of Web-based delivery systems.

**Trial Registration:**

ClinicalTrials.gov CT02739607; https://clinicaltrials.gov/ct2/show/study/NCT02739607 (Archived by WebCite at http://www.webcitation.org/6yY1VeYIZ)

## Introduction

### Background

The evidence-based approach to psychotherapy consists of a continuous effort devoted to explore the most effective strategies to reduce psychopathology used by researchers around the world. As support for the effectiveness of manualized, disorder-specific cognitive behavioral therapies (CBTs) emerged, some researchers geared their efforts toward gaining a broader understanding of psychopathology. Arguments for the high comorbidity of affective and anxiety disorders were fueled by the fact that about half of the clients diagnosed with one disorder also have a second diagnostic [1]. Such disorders present overlapping symptoms and share risk and maintaining factors, which further suggests a set of common higher order factors (ie, positive and negative emotions) across various Axis I diagnostic categories [2,3]. Barlow and colleagues [4] also convincingly argued that affective and anxiety disorders may be related to a number of common underlying mechanisms, while only trivial differences seem to separate them. This broader conceptualization of psychopathology inspired researchers and clinicians to develop transdiagnostic programs with common intervention components that match the higher order factors within a parsimonious and elegant framework [5-7]. Recently, numerous researchers joined this paradigmatic shift [8], planning [9] and publishing an amounting number of studies on this topic [10], and in 2017, the *Journal of Anxiety Disorders* dedicated a special issue to current and ongoing transdiagnostic approaches for anxiety.

As the literature evolved, the transdiagnostic label was used to cover conceptually dissimilar constructs. In an effort to gain a common understanding of this term, Sauer-Zavala and colleagues [11] suggested three classes of treatment approaches that are currently labeled as transdiagnostic: (1) universally applied therapeutic principles, (2) empirically based modular strategies, and (3) the shared mechanism approach. The universal therapeutic principles represent a *top-down* approach where general intervention techniques are used across disorders. For example, in the cognitive therapy framework, patients are encouraged to identify cognitive distortions and reevaluate experiences in a more realistic fashion, whereas in the acceptance and commitment therapy framework, patients are encouraged to be more accepting, to cultivate cognitive defusion, mindfulness, and to pursue their life values. The modular strategy represents an approach where relevant intervention strategies are used to address each problem presented by an individual patient regardless of his diagnostic. Finally, the shared mechanism approach focuses on addressing common underlying mechanisms according to theoretical models of psychopathology. For example, transdiagnostic interventions from this category (ie, Unified Protocol for Transdiagnostic Treatment of Emotional Disorders, UP) use a *bottom-up* approach by identifying the core vulnerabilities that contribute to the development and maintenance of multiple disorders and then design strategies to target them. However, research on the nature of shared mechanism or core underlying processes for affective and anxiety disorders is still emerging [12,13].

A number of individual studies have investigated the efficacy of transdiagnostic treatments, all with promising results [6,14]. In one of the largest randomized controlled trials (RCTs) of a transdiagnostic approach to date, Barlow and colleagues compared their UP with gold-standard, evidence-based protocols designed to treat the diagnostic-specific symptoms (ie, single-disorder protocols, SDPs) of generalized anxiety disorder (GAD), social anxiety disorder (SAD), obsessive compulsive disorder (OCD), and panic disorder/agoraphobia (PD/A). In this trial, the UP produced significant reductions in symptom severity across these disorders that were statistically equivalent to SDPs both at acute outcome and at 6-month follow-up [15]. In a series of 4 studies, Titov and colleagues [16-19] compared their transdiagnostic program with disorder-specific programs designed to address GAD, major depressive disorder (MDD), SAD, and PD/A, and those programs were delivered using either a clinician-guided or a self-guided format. Their results consistently showed equivalence between the two approaches (transdiagnostic vs disorder-specific) and the two treatment formats [16-19].

Recent reviews and meta-analytic studies also support the efficacy of transdiagnostic approaches. For example, after analyzing 11 studies where transdiagnostic interventions for various anxiety disorders were compared with wait-list control or treatment as usual, a moderate effect was revealed at posttreatment (*d*=0.68), which was maintained at follow-up [20]. The full spectrum of transdiagnostic packages for anxiety and affective disorders using a wide range of delivery methods (individual, group, computerized, or Internet supported) were included in a recent evaluation of 50 studies [21]. Here, large pre- to posttransdiagnostic treatment effect sizes (ESs) were found for depression (Hedges *g*=0.91) and anxiety (*g*=0.86) and moderate effects for quality of life (QoL; *g*=0.69). When the transdiagnostic treatments were compared with wait-list control or treatment as usual or attention training interventions, medium ES emerged for anxiety (*g*=0.65) and QoL (*g*=0.46), whereas large ES emerged for depression (*g*=0.80). Transdiagnostic and diagnostic-specific intervention programs for anxiety disorders were compared, and both program types yielded large ES and overlapping CIs [22]. Finally, a synthesis of 17 studies demonstrated that computerized or Internet-delivered transdiagnostic interventions outperformed their respective control groups on anxiety, depression, and QoL [23]. So it appears that the new generation of transdiagnostic programs are at least as effective as existing disorder-specific CBT protocols in reducing anxiety symptoms.

Advances in the evidence-based practice paradigm demand thorough investigations of intervention programs conducted in various cultures and contexts. Before being classified as highly effective, any new program should be validated by results obtained by at least two independent research teams [24]. Although previous research seems to favor transdiagnostic interventions for anxiety and depressive disorders, continued research is needed to further evaluate the efficacy of such programs around the world. To date, it is unclear how effective shared mechanism transdiagnostic programs are when used in various contexts and whether an abbreviated version delivered using a Web-based format represents a viable and effective treatment option. Moreover, combining the positive features of transdiagnostic programs with the advantages of Internet-delivered interventions (treatment fidelity, reduced costs, increased accessibility by disarming geographical barriers, and schedule conflicts) represents an important, innovative avenue for testing and disseminating evidence-based treatments.

### Study Aim

This study is part of the ongoing effort to explore the effectiveness of transdiagnostic programs as a way to strengthen the evidence-based approach to effective treatments [13,21,22]. Specifically, we evaluated the efficacy and acceptability of an established transdiagnostic treatment for anxiety and affective disorders [5] implemented in Romania using a Web-based guided delivery format. To our knowledge, this is the first evaluation of the UP using a Web-based format and at the same time the first time it was used on a Romanian sample. The UP was designed to address common underlying mechanism, and therefore, it pertains to the shared mechanism approach, being different from other transdiagnostic programs in terms of both design and components. Immediate and long-term (6 months) treatment effects were measured for a large sample of adults with anxiety and/or affective disorders that were randomized to either an active treatment condition or a wait-list control group. Our hypothesis was that participants receiving the study treatment would display significantly lower levels of depression and/or anxiety symptoms at the end of the treatment compared with those in the wait-list control group and that these improvements would be maintained 6 months following treatment. We also hypothesized that participants’ anxiety, sensitivity, and symptom interference would decrease, and their life quality would increase as a result of the treatment. Finally, we explored the impact of the intervention on participants’ perfectionism level, hoping to see a significant decrease on this emotion-driven behavior.

## Methods

### Overview

The study was approved by the Ethical Commission of West University of Timisoara, Romania (4509/26.02.2016) and was registered on ClinicalTrials.gov as NCT02739607. Written informed consent was obtained from all participants by surface mail.

### Participants

A total of 105 participants with a clinical diagnostic of either an affective disorder, an anxiety disorder, or any combination of affective and anxiety disorders were selected for this study. The online recruitment process was designed to be broadly inclusive, with few exclusion criteria. Eligibility and ineligibility criteria for participants are shown in Textboxes 1 and 2.

Individuals taking psychotropic medications at the time of enrollment were required to be stable on the same dose for at least 4 weeks before enrolling in the study. Furthermore, participants were asked neither to change their psychotropic medications nor to begin another psychosocial treatment program during the study.

**Eligibility criteria.**

Individuals were eligible for the study if they

1. were fluent in Romanian
2. were at least 18 years of age
3. had at least one self-report score within the cut-off range specified for each screening measure (eg, Beck Depression Inventory-II between 15-51, SPIN between 21-50, Penn State Worry Questionnaire between 45-68, YBOCS between 8-31, Panic Disorder Severity Scale-Self Report between 6-15, and *Posttraumatic stress disorder Checklist for* *Diagnostic and Statistical Manual of Mental Disorders, Fifth Edition* between 38-72)
4. received at least one current diagnostic of an affective (major depression or dysthymia) and/or an anxiety disorder on Structural Clinical Interview for Diagnostic and Statistical Manual of Mental Disorders, 4th. Edition Axis I Disorders
5. had no obstacle to participation (ie, had Internet access, did not have plans to travel for an extended time during the treatment, etc)

**Ineligibility criteria.**

Participants were excluded from the study if they

1. reported significant suicidal ideation (a score of 2 or above on the Beck Depression Inventory-II suicide item) or parasuicidal behavior (as measured by the Screening Questionnaire of the Structural Clinical Interview for Diagnostic and Statistical Manual of Mental Disorders, 4th Edition)
2. had one or more incompatible psychological disorders (ie, personality disorder, bipolar disorder, or psychosis)
3. displayed extremely high clinical symptoms (ie, over the highest specified cut-off score) on the self-report clinical measures (see also inclusion criteria 3)
4. currently receiving a psychosocial treatment

­

All excluded participants were directed toward other resources such as face-to-face psychotherapy and/or psychiatric assessment and treatment.

Participants who intended to remain anonymous were encouraged to create a special email account for this study. The Web platform did not allow multiple ID’s for the same email account. A confirmation massage was sent to the email account provided by the participant as a logistical measure.

### Study Procedure

The study was advertised in the local and national media following a press conference organized by the West University of Timisoara, Romania in April 2016. Interested participants could freely register or enroll for the study online [25]. After electronically signing the informed consent (ie, compulsory check box), participants were instructed to complete a series of online self-report measures as part of the screening process (see the Measures section). Participants who completed the online screening and scored in the range of the cut-off scores (mild to moderate clinical symptoms) were contacted and invited to take a phone interview using the Structural Clinical Interview for Diagnostic and Statistical Manual of Mental Disorders, 4th. Edition Axis I Disorders (SCID-I). The screening interviews (N=162) were conducted by graduate clinical psychology students, who were supervised by an experienced clinical psychologist. If participants met the diagnostic criteria for at least one affective and/or anxiety disorders, they were invited by email to take part in the study. At the end of the recruitment process, all registered participants received general feedback about their screening results. Excluded participants were informed about the reason for their status and guided to seek appropriate help in their community, whereas included participants were informed that they would be randomly assigned to either the immediate or the 10-week delayed treatment. Overall anxiety and depression symptom severity were collected online from all participants during the odd weeks of the program, while only the immediate treatment group received the transdiagnostic intervention. After the 10-week program, all included participants were invited to complete the postintervention online assessment and were contacted by phone to complete a diagnostic interview using the SCID-I. A different team of six graduate students blinded to participants’ preintervention diagnostic and group conducted the postintervention interviews under supervision. The postintervention online assessment included the same self-report measures as the screening, plus the *Treatment Satisfaction Questionnaire* for the active treatment group. After the postintervention assessment, the wait-list control group was invited to take part in the transdiagnostic treatment. Finally, a 6-month follow-up assessment was taken for the immediate-treatment group only, as the wait-list control group was lost to follow-up.

### The Transdiagnostic Treatment

In this study, participants received a Web-based transdiagnostic intervention for anxiety and mood disorders. The Romania version of the program was based on Barlow and colleague’s UP [5]. UP treatment modules were adapted for the online environment, but the treatment structure was conceptually similar. We retained only nine treatment sessions for our guided intervention, as our previous experience suggests this represents an adequate length for treating participants over the Internet [26]. In this study, a simplified treatment version was used with similar outcome results as the longer version [26]. The transdiagnostic program was designed as a stand-alone intervention, and the program content was unchanged during the trial. At the beginning of the treatment, participants were encouraged to complete one session per week and the associated homework assignments. The program first sought to increase participants’ motivation for the transdiagnostic treatment and guided them to define a set of specific treatment goals (session 1). Then participants were encouraged to intentionally notice their intense emotional experiences and to monitor them on a daily basis (session 2). Participant’s reaction to their intense emotions and a set of mindfulness exercises were addressed in the following week (session 3). The role of cognitive processes and the impact of cognitive distortions were presented next (session 4). The emotional avoidance and the concept of perfectionism as an instance of emotion-driven behavior (session 5) and the opposite action as a first attempt to practice exposure or behavioral activation (session 6) were presented next. Session 7 was entirely dedicated to confronting intense emotions via imaginary or in-vivo exposure, while session 8 continued the exposure process by guiding participants to confront their physical sensations. Finally, participants were asked to review the strategies learned throughout the entire program and to device a relapse prevention plan for the future (session 9). All participants were guided through the program in the same order (sessions 1-9), and access to the next session was granted if participants partially completed their homework assignments of the previous session.

Active treatment participants could access the nine sessions using their own device (computer, tablet, etc) at a time and place of their convenience. Eight graduate students in clinical psychology assisted and guided participants throughout the treatment by monitoring their activities on the platform and by exchanging written communications through an internal email system. Shortly before the study, the graduate students undertook an 8-hour training where the transdiagnostic concepts and principles were presented and exemplified by four case studies. Supervised by an experienced psychotherapist, the graduate students provided personalized feedback for participants’ homework assignments and answered their questions within a 24h interval. In terms of the frequency of message exchange, if participants did not initiate a written message with the graduate student assigned to them (which was seldom the case), they received a weekly feedback for their homework assignments. In case of inactivity, a participant received up to three written reminders on the platform, at a rate of one message per week. All participants were assisted free of charge for a 10-week interval: 9 weeks for each session and one extra week for their eventual delays. During this time, participants in the wait-list control group were only invited to complete the online self-report measures. After the 10-week treatment ended, no further guidance was provided, but participants continued to have access to the treatment for the next 6 months (until the follow-up assessment).

Our Web platform consists of two distinct but interconnected modules designed for online assessment and online psychotherapy. The Web platform was design as an infrastructure to facilitate written asynchronous communication. Access to the platform is controlled by ID and password, and all sensitive content is encrypted and stored on a secure server. The platform was previously tested during an open trial designed for healthy participants, and the only improvement consisted of the auto-save option for homework assignments. The Web platform was developed by a Romanian information technology team coordinated by the first author.

### Study Design, Randomization, and Power

To empirically investigate the efficacy of a Web-based transdiagnostic program, we used a (phase II) simple RCT design in which participants were assigned to either an immediate treatment or wait-list control group. Study design complied with the CONSORT EHEALTH checklist (see Multimedia Appendix 1). Randomization followed a 2:1 ratio, such that two-thirds of the participants were assigned to the immediate treatment group to maximize engagement and retain most participants for the postintervention assessment. The randomization was conducted with all included participants 1 day before starting the intervention program. One of the authors (AR), who was not involved in the selection process, generated the pseudorandom allocation sequence of participants’ ID by using an available online tool (https://www.randomizer.org). Technically, included participants were allocated to either the active treatment group or the wait-list control group by the graduate students working on the Web platform.

To estimate study power for two independent groups (one-tailed comparison), we used G*Power [27,28]. The RCT was powered to detect an ES of *d*=0.60 at posttreatment (Cronbach alpha=.05) at a power of 89% (1-beta). Although the 2:1 allocation ratio decrease study power, it was used to maximize participants’ involvement in the active treatment group.

### Outcome Measures

As the transdiagnostic intervention addressed simultaneously more than one disorder, more than one primary outcome measure was included. In our opinion, limiting the primary outcome measures to only one dimension would not have accurately reflected the outcomes of this trial.

#### Primary Outcome Measures

*Beck Depression Inventory-II* (BDI-II) [29] is a widely used 21-item measure of current depression. Data on the scale’s reliability and validty were reported in clinical samples [29,30], and the scale is considered an evidence-based outcome measure [31].

*Penn State Worry Questionnaire* (PSWQ) [32] is a 16-item measure of general anxiety or worry with excellent psychometric proprieties [33]. Compared with other anxiety disorders, PSWQ scores are higher for a GAD clinical sample [33,34], and the scale is considered an evidence-based outcome measure [35].

*Social Phobia Inventory* (SPIN) [36] is a 17-item measure design to assess participants’ social anxiety. The scale has good to excellent psychometric proprieties [36,37] and was considered an evidence-based outcome measure [38].

*Yale-Brown Obsessive Compulsive Scale* (Y-BOCS) [39] assesses the presence or severity of obsessions (items 1-5) and compulsions (items 6-10). The Y-BOCS has demonstrated good convergent validity, is sensitive to treatment-related change [39], and is considered an evidence-based outcome measure [40].

*Panic Disorder Severity Scale-Self Report* (PDSS-SR) [41] is a 5-item scale designed to capture panic symptoms. The PDSS-SR displays good internal consistency and construct validity [41].

*Posttraumatic stress disorder* (*PTSD) Checklist for Diagnostic and Statistical Manual of Mental Disorders-5th Edition* (PCL-5) [42] is a 20-item measure designed to assess participant’s level of posttraumatic stress. The scale demonstrated very good reliability and validity [42].

*Overall Anxiety Severity and Impairment Scale* (OASIS) [43] is a 5-item questionnaire developed to capture anxiety-related symptom severity and impairment across anxiety disorders. This measure has good to excellent psychometric proprieties [43,44].

*Overall Depression Severity and Impairment Scale* (ODSIS) [45] is also a 5-item instrument designed to measure severity and impairment of depressive symptoms. In a recent psychometric evaluation, the ODSIS demonstrated high internal consistency and good convergent and discriminant validity [45].

#### Secondary Outcome Measures

*Anxiety Sensitivity Index* (ASI) [46] is a 16-item questionnaire designed to assess fear of anxiety-related symptoms. The ASI displays a high internal consistency [46] and test-retest reliability [47] and is considered an evidence-based outcome measure [48].

*Quality of Life Inventory* (QOLI) [49] consists of 16 items pertaining life satisfaction. Respondents rate each item on its importance and overall satisfaction. Validity of the QOLI was demonstrated by positive correlations with other related measures of well-being and negative correlations with measures of psychopathology [49].

*Work and Social Adjustment Scale* (WSAS) [50] is a 5-item measure that captures the symptoms interference in work, home management, leisure, and family relationships. The WSAS has been successfully used in previous studies [51].

*Almost Perfect Scale-Revised* (APS-R) [52] is a 23-item questionnaire with three subscales: high standards, order, and discrepancy. Each item is rated on a 7-point scale, and higher scores indicate higher levels of perfectionism. The APS-R demonstrated good to excellent psychometric proprieties [52,53].

### Statistical Methods

Analyses were performed in SPSS version 24.0 (IBM Corp). Group differences in baseline characteristics were analyzed using the independent samples *t* test and the chi-square test. The postintervention and follow-up data were analyzed based on the intention-to-treat framework by the means of linear mixed models. This approach uses all available observations (maximum likelihood estimation) and allows for correlation between longitudinal data. For all the outcomes, time (baseline vs post intervention) was set as the within-group factor, and trial condition (transdiagnostic vs wait-list control group) was used as the between-groups factor. For the two outcomes that were also measured during the odd weeks of the treatment (ie, OASIS and ODSIS), the time factor had seven levels (ie, baseline, week 1, week 3, week, 5, week 7, week 9, and post intervention). We analyzed these two aforementioned variables both together with the entire set of outcomes (baseline vs post intervention comparison) to estimate the postintervention ES, and separately, to get a better grasp on the evolution of anxiety and depression symptoms during the treatment. Hence, we conducted separate analyses for each outcome with group, time, and group by time interaction as fixed effects and a random intercept for subject with an identity covariance structure. The group by time interactions express the mean change in outcomes from baseline to post treatment between the two trial groups. The fix effects of gender, previous psychotherapy experience in the past 4 years (dummy coded: yes or no), and treatment credibility were added to each model. Moreover, to investigate the long-term effects of the intervention on each outcome, and as the follow-up measures were collected only for the treatment group, we applied linear mixed models with time (baseline vs post treatment vs 6 months follow-up) as fixed factor and random intercept for subject. The fix effect of total number of homework assignments completed by participants (treatment adherence) was also added to each of these models. We made baseline to post treatment and baseline to follow-up comparisons to test the extent to which symptom amelioration was preserved in time.

We computed Hedges *g* effects size estimates for both between-groups and with-group comparisons. Between-groups comparisons were based on the mean differences from baseline to post treatment and the baseline SDs within each group. Within-group ESs were calculated for baseline to post treatment and for baseline to 6-month follow-up comparisons by correcting for the correlation between each pair of time points.

### Clinical Significance

To determine the clinical significance of this trial, we adopted the algorithm used by Ellard and colleagues [54] for both responder status and high end-state functioning. More precisely, at the post intervention, participants were considered *responders* if they evidenced a decrease of 30% or larger on at least two indicators from the following three measurement categories: (1) the specific diagnostic measure associated with their principal diagnostic (eg, BDI-II, PSWQ, SPIN, PDSS-SR, Y-BOCS, and PCL5), (2) the symptom interference measure (WSAS), or (3) the postintervention SCID-I interview (where they did not meet the diagnostic criteria for their principal disorder). The more stringent criteria for *high end-state functioning* involve the simultaneous fulfillment of two conditions: (1) not meeting diagnostic criteria for their principal diagnostic on the postintervention SCID-I interview and (2) displaying a subclinical score on either the WSAS or the disorder-specific measure associated with their principal disorder identified at baseline. Participants who changed psychotropic medication or started another treatment were excluded from these analyses.

## Results

### Participants’ Recruitment, Dropout, and Attrition

**Figure 1:**
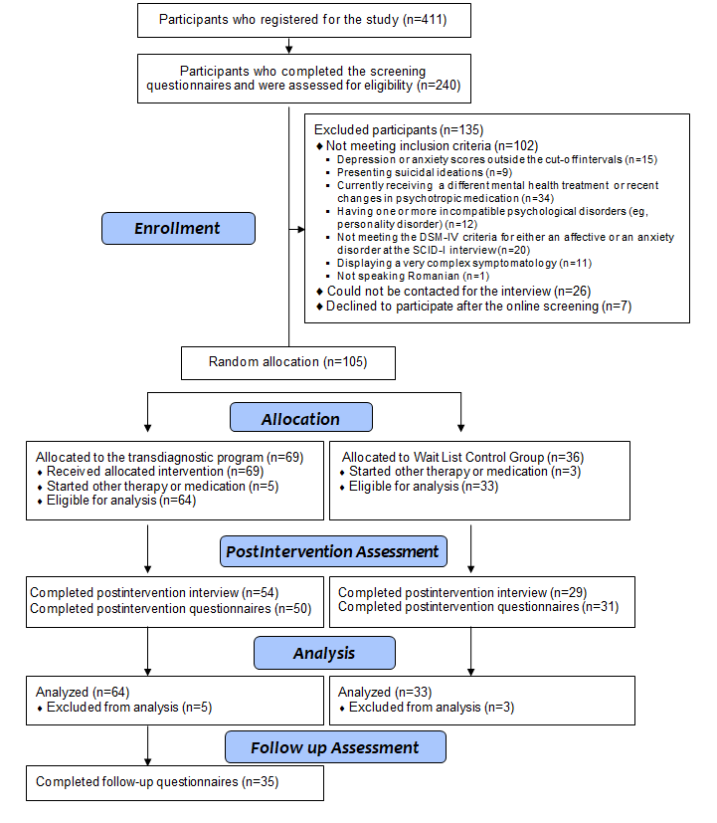
The flowchart depicting participants’ recruitment and progress throughout the program. SCID-I: Structural Clinical Interview for Diagnostic and Statistical Manual of Mental Disorders, 4th Edition Axis I Disorders.

Out of the 411 participants who expressed initial interest for the study, only 240 completed the online screening measures and were assessed for eligibility. Of those, 135 participants failed to meet initial study inclusion criteria. The 105 included participants were randomized into the treatment (N=69) and the control group (N=36). After the intervention, we conducted the SCID-I interview with 54 participants (78%, 54/69) from the active treatment group and with 29 participants (81%, 29/36) from the wait-list control group. A similar percentage of postintervention self-report measures were collected from both groups (see Figure 1). During the 10-week intervention program, 5 participants (7%, 5/69) from the treatment group and 3 (8%, 3/36) from the control group started or changed their psychotropic medication or started another psychosocial treatment.

**Figure 2:**
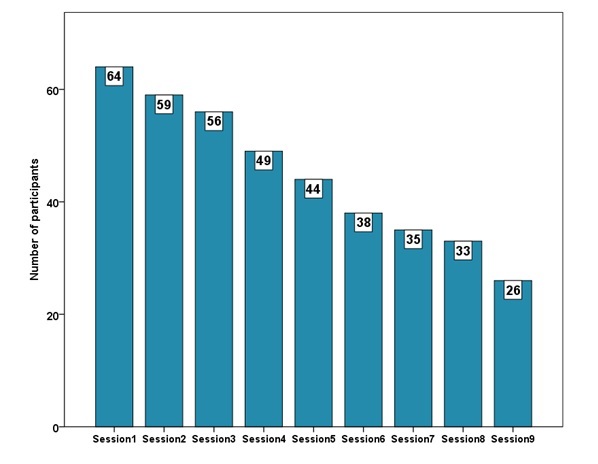
Attrition diagram.

Participants adherence during the program is illustrated in the attrition diagram (see Figure 2). Six-month follow-up assessment questionnaire were collected from 36 participants (52%, 36/69) in the active treatment group.

### Demographics and Baseline Clinical Status

Details regarding participants’ demographic characteristics are presented in Table 1. Participant’s overall mean age was 34.27 (SD 10.55, range 21-70), most of them having at least a college degree (44.2%, 46/105). The majority were females (80.9%, 85/105 overall). A lower proportion of males ended up in the wait-list control group compared with the active treatment group (*P*=.049). Therefore, we controlled for this variable in all subsequent analyses. Overall, 36.5% (38/105) received psychotherapy during the last 4 years. Even though not statistically significant (*P*=.07), there was a lower proportion of participants who benefited from psychotherapy in the previous 4 years in the active treatment group than in the wait-list control group. Hence, we also controlled for this factor in the main analyses. In all other respects, the treatment and control groups were similar in terms of demographic characteristics, including their time spend online.

Principal and comorbid clinical diagnostics are also presented in Table 1. Overall, disorders ranged between 1 and 5, with an average of 1.67 disorders per participant (SD 0.93).

#### Treatment Credibility

Both the active treatment group (mean 39.42, SD 8.40) and the wait-list control group (mean 36.42, SD 8.07) perceived the intervention as credible (the minimum and maximum possible scores range from 0-50), with no significant differences between the two groups (*t*_97_=1.72, *P*=.09).

#### Treatment Adherence

Treatment adherence was estimated using participants` online behavior: (1) how often they accessed the online treatment (number of logins) and (2) how often they were actively engaged with the content of the treatment (number of completed homework assignments—possible range 0-41). During the 10-week treatment period, the average number of platform accesses was 46.76 (SD 29.86) per participant (logins ranged between 2-149). Overall, the active treatment group participants completed 1355 homework assignments (mean 19.63, SD 12.10), and on average, participants completed 2.18 weekly assignments. Throughout the treatment, participants sent written messages to graduate students (mean 8.03, SD 6.78, range 0-28) and received written messages from them (mean 25.91, SD 9.38, range 8-44). At the end of the treatment, participants estimated to have spent an average 4 hours/week in treatment-related activities (SD 3.53; median 2.5 hours/week). As expected, a negative correlation was observed between the number of completed homework assignments and the number of disorders diagnosed after the treatment (*r*=−.23, *P=*.04).

### Effectiveness of the Intervention on Primary Outcomes

Table 2 includes the observed means and estimated marginal means for the primary outcomes measures. Linear mixed model results for the effectiveness of the intervention at post treatment and Hedges *g* ESs are displayed in Table 3. There were significant group by time interactions for BDI-II, PSWQ, and PCL-5 scores. SPIN scores were significantly reduced in the crude model, but the adjusted one drifted toward a trend (*P=*.05). The group by time interactions were nonsignificant for Y-BOCS and PDSS-SR, whereas the overall anxiety and depression measures (ODSIS and OASIS) yielded significant interaction effects. Hence, the transdiagnostic intervention was successful in reducing participants’ depression and anxiety, displaying between-group ESs that ranged from small (Hedges *g* was 0.39 for SPIN) to large (Hedges *g* was 0.86 for PCL-5).

**Figure 3:**
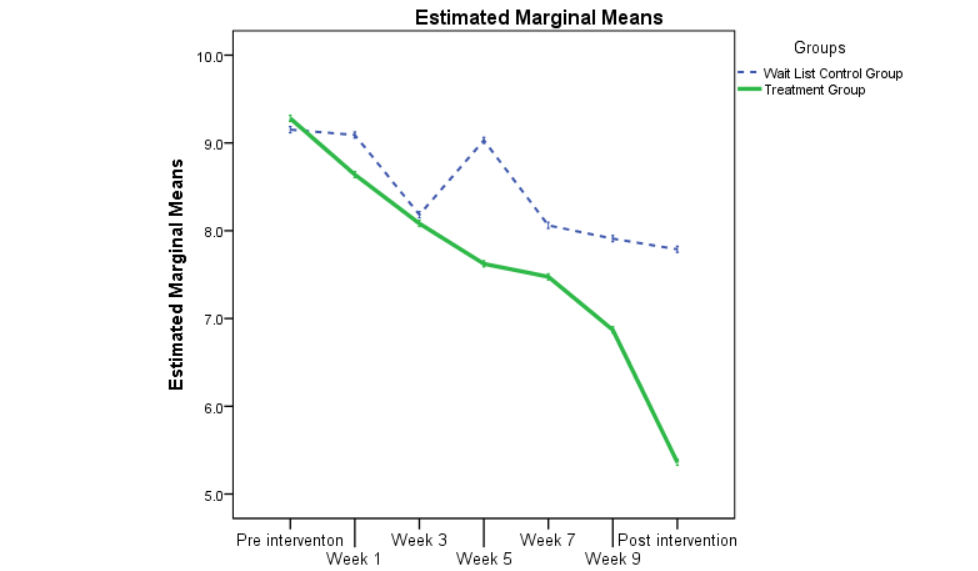
The evolution of the two groups during the treatment on the Overall Anxiety Severity and Impairment Scale (OASIS).

**Figure 4:**
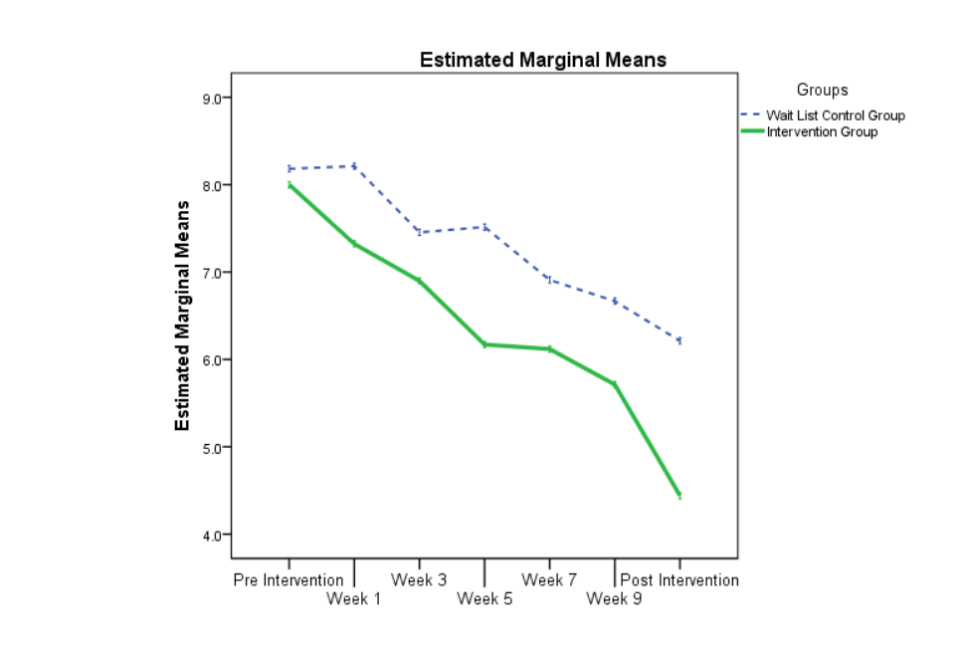
The evolution of the two groups during the treatment on the Overall Depression Severity and Impairment Scale (ODSIS).

The evolution of participants’ overall anxiety (OASIS) and overall depression (ODSIS) during the intervention is displayed in Figures 3 and 4. For both figures, intent to treat data was used (estimated marginal means). As can be intuitively visualized on the two graphs, the linear mixed model results revealed significant group by time interactions for both overall anxiety (*t*_399.94_=−4.09, *P*<.001) and overall depression (*t*_399.22_=−3.07, *P*=.002). More precisely, during the nine measurement occasion, there was a significant reduction in overall anxiety (mean difference −0.51, 95% CI −0.76 to −0.27) and overall depression (mean difference −0.44, 95% CI −0.73 to −0.16) for the treatment group participants as compared with those on the wait list.

### Effectiveness of the Intervention on Secondary Outcomes

Table 4 includes the descriptive statistics for the secondary outcomes, and Table 5 displays the group by time interaction results of the linear mixed models and the Hedges *g* ES estimates. Participants that benefited from the transdiagnostic intervention reported significant improvements from baseline to post treatment as compared with those in the control group regarding symptom interference (WSAS), anxiety sensitivity (ASI), and a trend for QoL (QOLI, *P*=.54), whereas the group by time interaction for perfectionism (APS-R) was nonsignificant. ESs were also medium to large (*g*=0.38 for QOLI and 0.80 for ASI). Additional analyses were conducted for Back Anxiety Inventory, Emotion Regulation Questionnaire, and Emotional Stability (see Multimedia Appendix 2).

**Table 1:** Baseline characteristics of the participants in the two groups and entire sample.

Variable		Active treatment group (n=69)	Wait-list control group (n=36)^a^	All participants (n=105)^a^	Statistic (*df*)	*P* value
**Age (years)**					*t* test=0.56 (102)	.57
	Mean (SD)	34.68 (11.63)	33.46 (8.1)	34.27 (10.55)		
	Range	21-70	21-60	21-70		
**Gender, n (%)**					Chi-square=3.8 (1)	.049
	Male	17 (25)	3 (9)	20 (19.2)		
	Female	52 (75)	32 (91)	84 (80.8)		
**Educational level, n (%)**					Chi-square=4.3 (5)	.49
	Postdoctoral studies	1 (1)	0 (0)	1 (1.0)		
	Doctoral degree	1 (1)	1 (3)	2 (1.9)		
	Master degree	18 (26)	11 (31)	29 (27.9)		
	Undergraduate degree	28 (41)	18 (51)	46 (44.2)		
	High school degree	19 (28)	5 (14)	24 (23.1)		
	Trade school degree	2 (3)	0 (0)	2 (1.9)		
**Marital status, n (%)**					Chi-square=2.7 (4)	.60
	Never married	26 (38)	16 (46)	42 (40.4)		
	In a relationship	15 (22)	6 (17)	21 (20.2)		
	Married	18 (26)	11 (31)	29 (27.9)		
	Divorced	8 (12)	2 (6)	10 (9.6)		
	Widowed	2 (3)	0 (0)	2 (1.9)		
**Principal diagnostic, n (%)**					Chi-square=7.5 (7)	.37
	GAD^b^	14 (20)	12 (33)	26 (24.8)		
	SAD^c^	18 (26)	5 (14)	23 (21.9)		
	MDD^d^	14 (20)	5 (14)	19 (18.1)		
	PD/A^e^	11 (16)	7 (20)	18 (17.2)		
	PTSD^f^	2 (3)	3 (8)	5 (4.7)		
	Specific phobia (SP)	2 (3)	2 (6)	4 (3.8)		
	OCD^g^	3 (4)	0 (0)	3 (2.8)		
	Other	5 (7)	2 (6)	7 (6.7)		
**Comorbid diagnostic, n (%)**					Chi-square=7.4 (7)	.38
	Any	35 (51)	19 (53)	54 (51.4)		
	GAD	8 (12)	4 (11)	12 (11.4)		
	SAD	13 (19)	2 (6)	15 (14.3)		
	MDD	8 (12)	8 (22)	16 (15.2)		
	PD/A	3 (4)	1 (3)	4 (3.8)		
	PTSD	0 (0)	1 (3)	1 (1)		
	SP	2 (3)	2 (6)	4 (3.8)		
	OCD	0 (0)	0 (0)	0 (0)		
	Other	1 (1)	1 (3)	2 (1.9)		
**Previous psychotherapy (in the last 4 years), n (%)**					Chi-square=3.2 (1)	.07
	Yes	21 (30)	17 (49)	38 (36.5)		
	No	48 (70)	18 (51)	66 (63.5)		
**Previous psychiatric diagnostic, n (%)**					Chi-square=0.0 (1)	.96
	Yes	20 (29)	10 (30)	30 (28.8)		
	No	49 (71)	25 (71)	74 (71.2)		
**Currently under medication, n (%)**					Chi-square=0.0 (1)	.98
	Yes	6 (9)	3 (9)	9 (8.7)		
	No	63 (91)	32 (91)	95 (91.3)		
**Time spent online (hours/day)**					*t* test=0.77 (102)	.44
	Mean (SD)	4.95 (3.77)	5.43 (3.41)	5.11 (2.99)		
	Range	1-13	1-15	1-15		

^a^One participant failed to complete the demographic questionnaire.

^b^GAD: generalized anxiety disorder.

^c^SAD: social anxiety disorder.

^d^MDD: major depressive disorder.

^e^PD/A: panic disorder/agoraphobia.

^f^Posttraumatic stress disorder.

^g^OCD: obsessive compulsive disorder.

### Primary Outcomes at Follow-Up

Within-group analyses for participants who received the transdiagnostic intervention revealed statistically significant improvements from baseline to post treatment for all the primary outcome measures (see Table 6). The reported ESs were also medium to large (*g* s: between 0.52 for Y-BOCS and 1.34 for OASIS). A similar pattern of results was found for the follow-up data. Treatment gains were generally maintained 6 months post intervention, with some measures displaying a further decrease at follow-up (ie, *g*=0.92 for SPIN).

### Secondary Outcomes at Follow-Up

Significant improvements from baseline to post treatment were reported for symptom interference (WSAS), QoL (QOLI), anxiety sensitivity (ASI), and discrepancy (APS-R; see Table 7). Most gains were maintained or improved 6 months following the intervention, except QOLI. Interestingly, after the program, participant’s high standards seem to decrease substantially (*P*=.02; *g*=0.32), and the ES for discrepancy increased from a small (*g*=0.25) to a medium effect (*g*=0.63).

### Clinical Significance

At the end of the treatment, 56% (22/69) participants from the active treatment group were classified as responders, compared with 17% (6/36) from the wait-list control group (χ^2^_1_=11.3, *P*=.001). Moreover, after the program, 27% (18/69) active treatment group participants were classified as being in a high end-state functioning compared with only 6% (2/36) from the wait-list control group (χ^2^_1_=7.3, *P*=.007). Data were also analyzed separately for reduction in the specific self-report measure associated with participants principal diagnostic (25%, 17/69 in the active treatment group vs 9%, 3/36 in the wait-list control group, χ^2^_1_=4.1, *P*=.04), for reduction in principal diagnostic (44%, 30/69 in the active treatment group vs 6%, 2/36 in wait-list control group, χ^2^_1_=15.3, *P*<.001), and for reductions in symptom interference (33%, 23/69 in the active treatment group vs 21%, 8/36 in wait-list control group, χ^2^_1_=1.8, *P*=.17). Finally, based on linear mixed models, we compared the reduction in the number of mental disorders from baseline to post treatment between the active intervention group and the wait-list control group. The group by time interaction was statistically significant: beta=−.92, 95% CI −1.35 to −0.49); *t*_90.78_=−4.25, *P*<.001; *g*=0.93, 95% CI0.50-1.35). Compared with the wait-list control group, participants in the active treatment group yielded a drop of almost one mental disorder as a result of the transdiagnostic intervention.

### Treatment Satisfaction

After the intervention, most treated participants declared to be satisfied or very satisfied with the program, displaying a mean score of 4.25 (SD 0.87) on a 5-point scale. They also declared that the information offered within the program was highly qualitative (mean 4.55, SD 0.77), and they considered the therapeutic-related activities as demanding (mean 2.75, SD 0.76). More importantly, most participants declared that the treatment helped them to better cope with their current difficulties (mean 3.34, SD 0.75). Finally, using a 10-point scale, participants declared that the transdiagnostic program appeared logical (mean 8.57, SD 1.99) and that they are confident to recommend it to someone facing similar difficulties (mean 8.38, SD 2.18).

**Table 2:** Observed means and estimated marginal means of the primary outcome measures at baseline, post intervention, and follow-up assessment.

Primary outcome		Observed means (SD)		Estimated means (standard error of mean)	
		Treatment^a^	Control^b^	Treatment (n=69)	Control (n=36)
**MDD^c^ (BDI-II^d^)**					
	Baseline	24.01 (11.71)	24.44 (12.33)	22.22 (1.65)	22.98 (2.29)
	Post intervention	10.81 (10.77)	19.52 (15.11)	8.93 (1.75)	19.63 (2.41)
	Follow-up	9.95 (9.27)	N/A	10.64 (1.55)	N/A^e^
**GAD^f^ (PSWQ^g^)**					
	Baseline	62.10 (9.39)	63.39 (12.67)	61.46 (1.57)	62.97 (2.17)
	Post intervention	53.53 (11.41)	60.68 (12.42)	52.14 (1.66)	61.42 (2.28)
	Follow-up	50.54 (10.75)	N/A	40.58 (1.25)	N/A
**SAD^h^ (SPIN^i^)**					
	Baseline	35.15 (15.15)	36.08 (13.95)	36.32 (2.02)	39.71 (2.80)
	Post-intervention	27.76 (13.74)	34.07 (14.12)	27.34 (2.16)	36.60 (2.96)
	Follow-up	24.00 (12.56)	N/A	21.99 (1.75)	N/A
**OCD^j^ (Y-BOCS^k^)**					
	Baseline	11.91 (8.55)	12.42 (9.00)	12.13 (1.13)	13.12 (1.57)
	Post intervention	7.87 (6.64)	9.55 (8.52)	7.71 (1.22)	11.17 (1.67)
	Follow-up	6.43 (5.71)	N/A	7.04 (1.11)	N/A
**PD/A^l^ (PDSS-SR^m^)**					
	Baseline	5.74 (5.85)	7.28 (6.77)	5.76 (0.78)	8.49 (1.08)
	Post intervention	2.31 (3.36)	5.58 (6.49)	2.63 (0.83)	6.79 (1.13)
	Follow-up	2.37 (4.22)	N/A	2.57 (0.76)	N/A
**PTSD^n^ (PCL-5^o^)**					
	Baseline	43.87 (13.97)	41.75 (15.97)	41.96 (2.18)	40.23 (3.03)
	Post intervention	25.74 (16.39)	33.28 (18.08)	22.41 (2.52)	33.47 (3.19)
	Follow-up	23.65 (15.63)	N/A	24.38 (2.47)	N/A
**Anxiety (OASIS^p^)**					
	Baseline	9.44 (3.88)	9.19 (4.21)	9.38 (0.56)	9.67 (0.78)
	Post intervention	4.52 (3.39)	7.44 (4.92)	4.33 (0.62)	7.83 (0.82)
	Follow-up	4.74 (3.94)	N/A	4.61 (0.61)	N/A
**Depression (ODSIS^q^)**					
	Baseline	8.53 (5.13)	8.25 (4.31)	7.99 (0.67)	7.96 (0.93)
	Post intervention	3.68 (3.78)	6.06 (5.62)	3.22 (0.74)	6.03 (0.98)
	Follow-up	4.23 (4.07)	N/A	4.25 (0.70)	N/A

^a^Unfortunately not all participants completed all self-report measures at postintervention and follow-up assessments. Therefore, the number of participants who provided postintervention data was as follows: 53 for MDD, GAD, SAD, and OCD; 51 for PD/A; 46 for PTSD; 50 for Anxiety; and 50 for Depression. The number of participants who provided follow-up data were as follows: 38 for MDD; 37 for GAD; 36 for SAD; 34 for PTSD; and 35 for OCD, PD/A, anxiety, and depression.

^b^The number of participants who completed postintervention data was 31 for MDD, GAD, SAD, OCD, and PD/A and 32 for PTSD, anxiety, and depression.

^c^MDD: major depressive disorder.

^d^BDI-II: Beck Depression Inventory-II.

^e^N/A: not applicable.

^f^GAD: generalized anxiety disorder.

^g^PSWQ: Penn State Worry Questionnaire.

^h^SAD: social anxiety disorder.

^i^SPIN: Social Phobia Inventory.

^j^OCD: obsessive compulsive disorder.

^k^Y-BOCS: Yale-Brown Obsessive Compulsive Scale.

^l^PD/A: panic disorder/agoraphobia.

^m^PDSS-R: Panic Disorder Severity Scale-Self Report.

^n^PTSD: posttraumatic stress disorder.

^o^PCL-5: PTSD Checklist for DSM-5.

^p^OASIS: Overall Anxiety Severity and Impairment Scale.

^q^ODSIS: Overall Depression Severity and Impairment Scale.

**Table 3:** Estimated differences in mean change of primary outcomes between baseline and post intervention for the transdiagnostic intervention group versus the wait-list control group.

Primary outcome and model (crude^a^ or adjusted^b^)		Estimate of mean change difference (95% CI)	*t* (*df*)	*P* value	Between-group Hedges *g* (95% CI)
**MDD^c^ (BDI-II^d^)**					
	Crude	−9.41 (−13.85 to −4.98)	−4.22 (85.11)	<.001	
	Adjusted	−9.94 (−14.51 to −5.37)	−4.33 (81.54)	<.001	0.83 (0.41-1.25)
**GAD^e^ (PSWQ^f^)**					
	Crude	−7.50 (−11.61 to −3.39)	−3.63 (87.98)	<.001	
	Adjusted	−7.76 (−12.04 to −3.49)	−3.61 (82.85)	.001	0.73 (0.31-1.14)
**SAD^g^ (SPIN^h^)**					
	Crude	−6.47 (−12.23 to −0.69)	−2.23 (85.00)	.02	
	Adjusted	−5.87 (−11.93 to 0.18)	−1.93 (80.30)	.05	0.39 (−0.01 to 0.80)
**OCD^i^ (Y-BOCS^j^)**					
	Crude	−2.23 (−5.58 to 1.13)	−1.32 (80.79)	.19	
	Adjusted	−2.47 (−5.95 to 1.01)	−1.41 (76.25)	.16	0.28 (−0.12 to 0.69)
**PD/A^k^ (PDSS-SR^l^)**					
	Crude	−1.58 (−3.59 to 0.43)	−1.56 (79.29)	.12	
	Adjusted	−1.44 (−3.54 to 0.66)	−1.37 (77.98)	.17	0.23 (−0.17 to 0.63)
**PTSD^m^ (PCL-5^n^)**					
	Crude	−12.58 (−19.73 to −5.44)	−3.50 (83.31)	.001	
	Adjusted	−12.79 (−20.24 to −5.35)	−3.42 (79.40)	.001	0.86 (0.45-1.28)
**Anxiety (OASIS^o^)**					
	Crude	−3.35 (−5.16 to −1.55)	−3.69 (86.02)	<.001	
	Adjusted	−3.20 (−5.07 to −1.33)	−3.40 (82.17)	.001	0.80 (0.38-1.21)
**Depression (ODSIS^p^)**					
	Crude	−2.69 (−4.67 to −0.73)	−2.73 (80.64)	.008	
	Adjusted	−2.85 (−4.88 to −0.82)	−2.79 (77. 63)	.006	0.58 (0.17-0.99)

^a^Crude model: raw association (without being adjusted for supplementary covariates).

^b^Adjusted model: adjusted for gender, previous psychotherapy experience in the past 4 years (dummy coded: yes or no), and treatment credibility.

^c^MDD: major depressive disorder.

^d^BDI-II: Beck Depression Inventory-II.

^e^GAD: generalized anxiety disorder.

^f^PSWQ: Penn State Worry Questionnaire.^g^SAD: social anxiety disorder.

^h^SPIN: Social Phobia Inventory.

^i^OCD: obsessive compulsive disorder.

^j^Y-BOCS: Yale-Brown Obsessive Compulsive Scale.

^k^PD/A: panic disorder/agoraphobia.

^l^PDSS-SR: Panic Disorder Severity Scale-Self Report.

^m^PTSD: posttraumatic stress disorder.

^n^PCL-5: PTSD Checklist for DSM-5.

^o^OASIS: Overall Anxiety Severity and Impairment Scale.

^p^ODSIS: Overall Depression Severity and Impairment Scale.

**Table 4:** Observed means and estimated marginal means of the secondary outcome measures at baseline, post intervention, and follow-up assessment.

Secondary outcome			Observed mean (SD)		Estimated mean (standard error of mean)	
			Treatment^a^	Control^b^	Treatment (n=69)	Control (n=36)
**Symptom interference (WSAS^c^)**						
	Baseline		19.38 (9.00)	18.97 (9.55)	18.32 (1.25)	17.95 (1.73)
	Post intervention		11.90 (8.83)	14.97 (9.30)	10.57 (1.39)	14.61 (1.82)
	Follow-up		11.06 (8.59)	N/A^d^	11.44 (1.41)	N/A
**Quality of life (QOLI^e^)**						
	Baseline		0.45 (1.82)	0.78 (1.82)	0.48 (.25)	0.92 (0.35)
	Post intervention		1.35 (1.73)	1.03 (1.77)	1.24 (0.28)	0.99 (0.36)
	Follow-up		1.03 (1.69)	N/A	1.01 (0.26)	N/A
**Anxiety sensitivity (ASI^f^)**						
	Baseline		29.81 (11.18)	31.71 (12.43)	30.85 (1.62)	34.29 (2.25)
	Post intervention		19.86 (12.15)	29.36 (12.81)	19.45 (1.80)	32.24 (2.38)
	Follow-up		14.77 (8.42)	N/A	15.51 (1.59)	N/A
**Perfectionism (APS-R^g^)**						
	**High standards**					
		Baseline	38.62 (7.14)	40.47 (5.46)	38.17 (0.93)	40.11 (1.29)
		Post intervention	38.74 (6.41)	39.28 (6.14)	37.92 (1.02)	39.22 (1.35)
		Follow-up	36.31 (6.52)	N/A	36.56 (1.02)	N/A
	**Order**					
		Baseline	20.12 (5.69)	20.00 (4.93)	20.60 (0.78)	20.51 (1.09)
		Post intervention	21.16 (4.91)	19.25 (5.94)	20.76 (0.82)	19.72 (1.11)
		Follow-up	20.94 (4.93)	N/A	19.88 (0.77)	N/A
	**Discrepancy**					
		Baseline	58.80 (15.81)	57.22 (17.14)	58.53 (2.44)	59.44 (3.83)
		Post intervention	55.02 (19.52)	51.66 (16.43)	53.56 (2.64)	53.32 (3.52)
		Follow-up	49.86 (15.26)	N/A	48.57 (2.52)	N/A

^a^Unfortunately not all participants completed all self-report measures at postintervention and follow-up assessments. Therefore the number of participants who provided postintervention data was as follows: 50 for symptom interference; 46 for quality of life; 49 for anxiety sensitivity, and for perfectionism; and 35 participants provided follow-up data for all secondary outcomes.

^b^The number of participants who completed postintervention data was as follows: 32 for symptom interference and perfectionism; 30 for quality of life; and 31 for anxiety sensitivity.

^c^WSAS: Work and Social Adjustment Scale.

^d^N/A: not applicable.

^e^QOLI: Quality of Life Inventory.

^f^ASI: Anxiety Sensitivity Index.

^g^APS-R: Almost Perfect Scale-Revised.

**Table 5:** Estimated differences in mean change of secondary outcomes between baseline and post intervention for the transdiagnostic intervention group versus the wait-list control group.

Secondary outcome and model (crude^a^ or adjusted^b^)			Estimate of mean change difference (95% CI)	*t* (degrees of freedom)	*P* value	Between-group Hedges *g* (95% CI)
**Symptom interference (WSAS^c^)**						
	Crude		−4.13 (−8.14 to −0.13)	−2.05 (82.03)	.04	
	Adjusted		−4.41 (−8.48 to −0.35)	−2.16 (81.16)	.03	0.48 (0.07-0.88)
**Quality of life (QOLI^d^)**						
	Crude		0.73 (0.04-1.41)	2.11 (78.44)	.03	
	Adjusted		0.69 (−0.01 to 1.40)	1.96 (74.35)	.05	0.38 (−0.03 to 0.78)
Anxiety sensitivity (ASI^e^)						
	Crude		−9.09 (−13.91 to −4.27)	−3.76 (79.54)	<.001	
	Adjusted		−9.35 (−14.26 to −4.44)	−3.79 (78.06)	<.001	0.80 (0.38-1.22)
**Perfectionism (APS-R)**						
	**High standards**					
		Crude	0.62 (−1.99 to 3.22)	0.47 (82.17)	.63	
		Adjusted	0.64 (−2.06to 3.34)	0.47 (79.31)	.64	−0.10 (−0.50 to 0.31)
	**Order**					
		Crude	0.96 (−0.49 to 2.40)	1.32 (77.38)	.19	
		Adjusted	0.94 (−0.54 to 2.41)	1.26 (75.42)	.21	−0.17 (−0.58 to 0.23)
	**Discrepancy**					
		Crude	0.32 (−6.12 to 6.76)	0.09 (83.51)	.92	
		Adjusted	1.15 (−5.48 to 7.79)	0.35 (80.21)	.73	−0.07 (−0.47 to 0.33)

^a^Crude model: raw association (without being adjusted for supplementary covariates).

^b^Adjusted model: adjusted for gender, previous psychotherapy experience in the past 4 years (dummy coded: yes or no), and treatment credibility.

^c^WSAS: Work and Social Adjustment Scale.

^d^QOLI: Quality of Life Inventory.

^e^ASI: Anxiety Sensitivity Index.

^f^APS-R: Almost Perfect Scale-Revised.

**Table 6:** Within-group estimated changes in primary outcomes for the active treatment group. All estimates are adjusted for treatment adherence (total number of homework assignments completed by participants).

Primary outcome		*F* (*df*)	*P* value	Estimate (95% CI)	*P* value	Within-group Hedges *g* (95% CI)
**MDD^a^ (BDI-II^b^)**						
	Time	53.23 (2,88.82)	<.001			
	Baseline vs post test			−13.09 (−15.89 to −10.29)	<.001	1.18 (0.85-1.51)
	Baseline vs follow-up			−12.66 (−15.78 to −9.53)	<.001	1.17 (0.69-1.65)
**GAD^c^ (PSWQ^d^)**						
	Time	33.80 (2,95.69)	<.001			
	Baseline vs post test			−8.96 (−11.64 to −6.28)	<.001	0.85 (0.54-1.15)
	Baseline vs follow-up			−11.01 (−14.02 to−7.99)	<.001	1.06 (0.67-1.46)
**SAD^e^ (SPIN^f^)**						
	Time	23.13 (2,90.19)	<.001			
	Baseline vs post test			−8.96 (−12.59 to −5.33)	<.001	0.61 (0.31-0.91)
	Baseline vs follow-up			−13.24 (−17.38 to −9.09)	<.001	0.92 (0.54-1.31)
**OCD^g^ (Y-BOCS^h^)**						
	Time	10.79 (2,83.17)	<.001			
	Baseline vs post test			−4.03 (−6.02 to −2.03)	<.001	0.52 (0.23-0.81)
	Baseline vs follow-up			−4.39 (−6.69 to −2.09)	<.001	0.58 (0.19-0.95)
**PD/A^i^ (PDSS-SR^j^)**						
	Time	13.67 (2,72.75)	<.001			
	Baseline vs post test			−3.24 (−4.59 to −1.88)	<.001	0.65 (0.31-0.99)
	Baseline vs follow-up			−3.06 (−4.59 to −1.52)	<.001	0.58 (0.17-0.99)
**PTSD^k^ (PCL-5^l^)**						
	Time	33.53 (2,88.28)	<.001			
	Baseline vs post test			−18.43 (−23.59 to −13.25)	<.001	1.22 (0.77-1.67)
	Baseline vs follow-up			−18.93 (−24.59 to −13.27)	<.001	1.25 (0.65-1.84)
**Anxiety (OASIS^m^)**						
	Time	39.05 (2,92.25)	<.001			
	Baseline vs post test			−4.93 (−6.16 to −3.69)	<.001	1.34 (0.95-1.74)
	Baseline vs follow-up			−4.72 (−6.10 to −3.35)	<.001	1.18 (0.61-1.75)
**Depression (ODSIS^n^)**						
	Time	29.28 (2,81.84)	<.001			
	Baseline vs post test			−4.67 (−5.98 to −3.37)	<.001	1.00 (0.65-1.35)
	Baseline vs follow-up			−4.08 (−5.53 to −2.62)	<.001	0.85 (0.44-1.27)

^a^MDD: major depressive disorder.

^b^BDI-II: Beck Depression Inventory-II.

^c^GAD: generalized anxiety disorder.

^d^PSWQ: Penn State Worry Questionnaire.

^e^SAD: social anxiety disorder.

^f^SPIN: Social Phobia Inventory.

^g^OCD: obsessive compulsive disorder.

^h^Y-BOCS: Yale-Brown Obsessive Compulsive Scale.

^i^PD/A: panic disorder/agoraphobia.

^j^PDSS-R: Panic Disorder Severity Scale-Self Report.

^k^PTSD: posttraumatic stress disorder.

^l^PCL-5: PTSD Checklist for DSM-5.

^m^OASIS: Overall Anxiety Severity and Impairment Scale.

^n^ODSIS: Overall Depression Severity and Impairment Scale.

**Table 7:** Within-group estimated changes in secondary outcomes for the active treatment group. All estimates are adjusted for treatment adherence (total number of homework assignments completed by participants).

Secondary outcome			*F* (*df*)	*P* value	Estimate (95% CI)	*P* value	Within-group Hedges *g* (95% CI)
**Symptom interference (WSAS^a^)**							
	Time		19.98 (2,84.19)	<.001			
	Baseline vs post test				−7.25 (−9.90 to −4.61)	<.001	0.79 (0.46-1.13)
	Baseline vs follow-up				−7.75 (−10.69 to −4.79)	<.001	0.86 (0.43-1.29)
**Quality of life (QOLI^b^)**							
	Time		5.50 (2,83.04)	.006			
	Baseline vs post test				0.69 (0.28-1.12)	.001	0.39 (0.13-0.64)
	Baseline vs follow-up				0.39 (−0.07 to 0.85)	.10	0.21 (−0.04 to 0.47)
**Anxiety sensitivity (ASI^c^)**							
	Time		55.30 (2,81.78)	<.001			
	Baseline vs post test				−11.17 (−13.88 to −8.45)	<.001	1.00 (0.69-1.32)
	Baseline vs follow-up				−14.25 (−17.25 to −11.25)	<.001	1.38 (0.91-1.86)
**Perfectionism (APS-R^d^)**							
	**High standards**						
		Time	2.99 (2,85.85)	.05			
		Baseline vs post test			−.37 (−2.07 to 1.34)	.66	0.05 (−0.21 to 0.31)
		Baseline vs follow-up			−2.26 (−4.16 to −.36)	.02	0.32 (0.02-0.63)
	**Order**						
		Time	.69 (2, 81.40)	.50			
		Baseline vs post test			0.09 (−0.90 to 1.09)	.85	−0.02 (−0.21 to 0.17)
		Baseline vs follow-up			−0.54 (−1.65 to 0.57)	.33	−0.09 (−0.13 to 0.33)
	**Discrepancy**						
		Time	9.40 (2,86.06)	<.001			
		Baseline vs post test			−4.62 (−8.75 to −.49)	.03	0.25 (0.02-0.48)
		Baseline vs follow-up			−9.99 (−14.59 to −5.39)	<.001	0.63 (0.30-0.96)

^a^WSAS: Work and Social Adjustment Scale.

^b^QOLI: Quality of Life Inventory.

^c^ASI: Anxiety Sensitivity Index.

^d^APS-R: Almost Perfect Scale-Revised.

## Discussion

### Principal Findings

The broader transdiagnostic conceptualizations of emotional disorders have recently attracted researchers’ attention as a promising and parsimonious approach to treatment [11-13,15,21,23,55]. This study was designed to examine the efficacy and acceptability of an Internet-delivered, clinician-assisted transdiagnostic program for depression and anxiety disorders in Romania. The broadly inclusive intake criteria allowed us to select 105 participants with a principal diagnostic of MDD, GAD, SAD, PD/A, OCD, and PTSD, with more than half of them (51.4%, 54/105) having at least a second clinical diagnostic.

As predicted, the transdiagnostic program led to significantly greater reductions (relative to the wait-list control group) in symptom severity across both principal and comorbid disorders, as well as significant decreases in functional impairment that were maintained over time. Furthermore, participants receiving the treatment evidenced greater rates of recovery on several symptom measures (except Y-BOCS and PDSS-SR), and were less likely to meet criteria for mental disorders following treatment. Moderate to large ES estimates were obtained for most diagnostic-specific measures (ie, BDI-II, PSWQ, SPIN, and PCL-5), as well as for the general measures of anxiety and depression (OASIS and ODSIS), suggesting that transdiagnostic treatments may be effective across a range of affective and anxiety disorders. Our effects were comparable (overlapping 95% CI) with those reported in other transdiagnostic trials [6,15].

The between-group insignificant differences and small ES for OCD and PD/A could be explained on the one hand by the small number of participants meeting diagnostic criteria for OCD (N=3) and on the other hand by the measurement choice for PD/A (ie, PDSS-SR was *not* classified as an evidence-based measure [48]. Moreover, Erickson and colleagues mentioned that OCD participants seem to be less motivated and more complex cases, distracting people with other anxiety disorders participating in a group transdiagnostic program [56]. Finally, after comparing several Web-based intervention programs [57], only a trend decrease for the PD/A diagnostic number was observed, whereas all other anxiety disorders (ie, GAD and SAD) yielded significant results. A larger study that allows differential efficacy comparison between primary diagnostics could further clarify this issue.

Relative to the wait-list control group, our transdiagnostic intervention also led to significantly greater improvements in symptom interference, anxiety sensitivity, emotional stability, QoL (at trend level), and one component of emotion regulation. Improvements in this area are not particularly surprising given the focus of the program on emotion and the development of emotion regulation skills, but is encouraging to see nevertheless. Anxiety sensitivity, conceptualized as the fear of bodily sensations, was first associated with panic disorder. However, recent research has demonstrated that it may be common across emotional disorders [58,59]. Results from this study replicate those from Boswell and colleagues providing additional support for the transdiagnostic relevance of anxiety sensitivity [58].

The other transdiagnostic process—perfectionism—displayed consistent within-group improvements 6 month after the intervention, when both high standards and discrepancy subscales significantly decreased. Although our transdiagnostic program explicitly addressed perfectionism as an instance of emotion-driven behavior, it seems that the effects of the intervention were mostly visible on the long term for perfectionism. In another intervention program conducted by our group, we found a significant decrease in perfectionism following a 45-day intervention [60]. Despite the fact that the aforementioned program was designed to comprehensively address perfectionism in nine sessions, only a small percentage of participants displayed a decrease of more than 50% from their initial perfectionism level (recovery rates between 0%-4.9%), whereas higher impact was observed for associated levels of depression and anxiety (recovery rates between 14.6%-31.7%) [60]. Such results are in line with previous literature supporting the trait-like stability of perfectionism [61].

### Treatment Satisfaction

The overall treatment satisfaction was high; participants acknowledging to have received qualitative information related to their disorders and to be better equipped to cope with their current difficulties. The treatment approach appeared logical to most participants, and they seemed willing to recommend the transdiagnostic program to other people with similar problems. This suggests that the program appears useful to participants and could eventually be implemented to similar samples of internet users outside of the present trial. One potentially positive feature that could be added in clinical practice is a short (bimonthly) telephone contact that could contribute to solving simple problems and facilitate participant involvement.

### Advantages of Web-Based Transdiagnostic Interventions

The transdiagnostic programs definitely represent a successful approach to treatment as they are better designed to address comorbidity. Transdiagnostic programs are broadly inclusive and can elegantly address multiple problems in a parsimonious manner [15]. It was previously reported that participants seemed interested to find out more about symptoms and coping strategies that are not directly related to their current difficulties, but represent core underlying mechanisms for multiple disorders [62,63]. Moreover, the Web-based format of our intervention allowed successful administration of the program with only a brief therapist training. In addition, it is generally accepted that Web-based programs are less time-intensive for practitioners, as some of the therapy tasks (ie, explaining the relationships between thoughts, feelings, and behaviors) are carried out by the computerized system, and human effort is directed toward more complex therapy tasks (ie, assisting each participant and offering personalized feedback) (see Andersson [64]). Moreover, by providing remote access to the program, participants from rural areas could easily access the program despite the scarcity of available clinicians in their neighborhood [65].

### Study Limitations

Finally, our results should be considered in light of several limitations. First, although this study included patients with a range of affective and anxiety disorders, it was not adequately powered to investigate the differential efficacy of the transdiagnostic program for each primary diagnostic category, particularly in the wait-list control group. Therefore, we could not meaningfully compare the effects of the program as a function of principal diagnostic, but this might be an important question for future studies. Second, we excluded participants with very complex symptoms (N=11), and the mean number of baseline diagnostics per participant were somehow smaller in our sample (1.67) compared with other trials (eg, 2.3 comorbid diagnostic [15]). This represents an inherent limitation associated with the treatment delivery format, as it was argued that Web-based programs may not be sufficient for the most severe cases [66]. Third, only half of the participants in the active treatment condition completed the follow-up questionnaires. Although such dropout rates are not uncommon in Web-based studies [67], the results should be interpreted with caution as this might favor treatment effects overestimations. Fourth, a relatively high attrition rates was observed in our trial, as only 44/69 participants completed at least five sessions, and only 23/69 completed all nine sessions. This is somehow surprising considering that we included a motivational interviewing in session one and used a 2:1 ratio to offer a greater number of participants the possibility to start the treatment immediately after the initial assessment. It is possible that factors related to participants’ characteristics (ie, expectation to have a Skype-like interaction with the therapists) could have played a role in the initial adherence, whereas other factors (ie, treatment workload) could have played a role in the subsequent involvement with the program. Moreover, a possible confound is that participants who finished all or most of the treatment sessions were (over)motivated to seek treatment before it started. However, to disentangle the factors that play a significant role in the adherence process, future study should consider a broader conceptualization of this process that involves therapy-related factors, patient-related factors, disorder-related factors, and socioeconomic and health-system factors [68]. In this context, low adherence could represent a mismatch between one or more of the aforementioned factors (see also [69,70]).

### Study Summary

Summarizing, efficacious treatments for affective, anxiety, and related disorders exist [23,55,71-73], but implementing them in other contexts and cultures is still limited [74]. The transdiagnostic intervention tested in this study offers a treatment option that capitalizes on the shared mechanisms approach, with an increased dissemination potential through the use of a Web-based delivery system. This format minimizes direct clinician involvement, which greatly reduces one of the primary barriers to dissemination of empirically supported psychological treatments; namely, training community clinicians to utilize these treatments effectively and with fidelity. Overall, the results of this study also provide further support for the efficacy and acceptability of transdiagnostic evidence-based treatments targeting emotion (dys)regulation. Further research evaluating such programs, particularly in community settings, is needed.

## Appendix

Multimedia Appendix 1 CONSORT‐EHEALTH checklist (V 1.6.1).

Multimedia Appendix 2 Supplementary analysis.

## Abbreviations

APS-R: Almost Perfect Scale-Revised
ASI: Anxiety Sensitivity Index
BDI-II: Beck Depression Inventory-II
CBT: cognitive behavioral therapy
ES: effect size
GAD: generalized anxiety disorder
MDD: major depressive disorder
OCD: obsessive compulsive disorder
OASIS: Overall Anxiety Severity and Impairment Scale
ODSIS: Overall Depression Severity and Impairment Scale
PCL-5: PTSD Checklist for DSM-5
PD/A: panic disorder/agoraphobia
PDSS-SR: Panic Disorder Severity Scale-Self Report
PSWQ: Penn State Worry Questionnaire
PTSD: posttraumatic stress disorder
QoL: quality of life
QOLI: Quality of Life Inventory
RCT: randomized controlled trial
SAD: social anxiety disorder
SCID-I: Structural Clinical Interview for DSM-IV
SDP: single-disorder protocol
SPIN: Social Phobia Inventory
UP: unified protocol
WSAS: Work and Social Adjustment Scale
Y-BOCS: Yale-Brown Obsessive Compulsive Scale
