# Supplementary material for: A Web-Based Transdiagnostic Intervention for Affective and Mood Disorders: Randomized Controlled Trial
Source: JMIR Ment Health. 2018 May 24;5(2):e36. doi: 10.2196/mental.8901 (PMC5992454; doi:10.2196/mental.8901)
Supplement: Multimedia Appendix 2 [file mental_v5i2e36_app2.pdf]

Multimedia Appendix 2 Supplementary analysis

Table 8. Observed means and estimated marginal means of the secondary outcome measures at baseline, post-intervention and follow-up assessment.

Measure	Observed means (SD)		Estimated means (SEM)	
	Treatment	Control	Treatment (n = 69)	Control (n = 36)
**Anxiety (BAI[a])**				
Baseline	24.52 (12.54)	28.50 (14.02)	23.93 (1.60)	29.89 (2.22)
Post-intervention	14.06 (8.00)	21.13 (11.98)	13.59 (1.79)	22.78 (2.34)
Follow-up	12.40 (8.78)	-	12.46 (1.72)	-
**Emotional stability (IPIP[b])**				
Baseline	19.59 (6.35)	20.00 (7.19)	20.32 (1.02)	21.52 (1.43)
Post-intervention	24.38 (8.63)	21.72 (6.93)	25.71 (1.12)	22.51 (1.48)
Follow-up	26.06 (7.09)	-	26.01 (1.15)	-
**Emotion regulation (ERQ[c])**				
Cognitive reappraisal				
Baseline	26.69 (7.01)	25.83 (5.42)	26.32 (0.93)	25.67 (1.29)
Post-intervention	28.54 (6.59)	24.34 (7.05)	28.39 (1.04)	24.42 (1.36)
Follow-up	28.69 (5.76)	-	28.35 (1.01)	-
Expressive suppression				
Baseline	16.48 (6.11)	15.71 (6.15)	17.26 (0.81)	17.39 (1.12)
Post-intervention	14.48 (5.81)	14.75 (5.78)	15.18 (0.88)	16.65 (1.17)
Follow-up	14.23 (5.70)	-	13.26 (0.87)	-

[a] BAI: Back Anxiety Inventory
[b] IPIP
[c] ERQ: Emotion Regulation Questionnaire

Table 9. Within-group estimated changes in outcome measures for the active treatment group[a]

Measure [b]	Model	Estimate of mean change difference (95% CI)	*t (df)*	*P*	Between-group Hedges's *g* (95% CI)
**Anxiety (BAI)**	Crude	-3.79 (-9.02, 1.43)	-1.45 (80.69)	.152	
	Adjusted	-3.22 (-8.59, 2.16)	-1.19 (79.79)	.237	.25 (-.16, .65)
**Emotional stability (IPIP)**	Crude	4.20 (1.17, 7.24)	2.76 (83.67)	.007	
	Adjusted	4.41 (1.30, 7.51)	2.83 (80.12)	.006	.66 (.24, 1.07)
**Emotion regulation (ERQ)**					
Cognitive reappraisal	Crude	3.31 (0.10, 6.52)	2.05 (84.19)	.043	
	Adjusted	3.32 (0.03, 6.61)	2.01 (80.77)	.048	.51 (.10, .91)
Expressive suppression	Crude	-1.44 (-3.69, 0.81)	-1.27 (81.35)	.206	
	Adjusted	-1.34 (-3.65, 0.97)	-1.16 (78.27)	.251	.22 (-.19, .62)

[a] All estimates are adjusted for treatment adherence (total number of homework assignments completed by participants).
[b] The abbreviations for all primary outcomes are mentioned at the end of Table 8.

Table 10. Within-group estimated changes in outcome measures for the active treatment group[a]

Measure [b]	*F (df)*	*P*	Estimate (95% CI)	*P*	Within-group Hedges's *g* (95% CI)
**Anxiety (BAI)**					
Time	23.75 (2, 78.11)	< .001			
Baseline vs. Post-test			-10.72 (-14.39, -7.04)	< .001	1.01 (0.57, 1.44)
Baseline vs. Follow-up			-11.89 (-15.98, -7.82)	< .001	1.08 (0.49, 1.65)
**Emotional stability (IPIP)**					
Time	16.08 (2, 90.30)	< .001			
Baseline vs. Post-test			5.09 (2.88, 7.29)	< .001	.65 (.35, .96)
Baseline vs. Follow-up			6.14 (3.69, 8.59)	< .001	.89 (.41, 1.38)
**Emotion regulation (ERQ)**					
Cognitive reappraisal					
Time	1.59 (2, 83.53)	.211			
Baseline vs. Post-test			1.65 (-0.27, 3.57)	.091	.25 (-.05, .54)
Baseline vs. Follow-up			1.26 (-0.88, 3.40)	.244	.19 (-.15, .53)
Expressive suppression					
Time	10.55 (2, 83.68)	< .001			
Baseline vs. Post-test			-2.07 (-3.38, -0.76)	.002	.34 (.09, .59)
Baseline vs. Follow-up			-3.21 (-4.68, -1.75)	< .001	.48 (.22, .74)

[a] All estimates are adjusted for treatment adherence (total number of homework assignments completed by participants).

[b] The abbreviations for all secondary outcomes are mentioned at the end of Table 8.
